# News and Events of the Week

**Published:** 1910-09-10

**Authors:** 


### News and Events of the Week.

The Huxley Lecture will be delivered in the Out-patients' Hall at Charing Cross Hospital, by Dr. F. W. Mott, F.R.S., on Monday, October 3, 1910, at 4.30 p.m.

At the Coventry Police Court recently Samuel Robinson, who had been remanded on the charge of stealing a hospital donation-box from one of the public-houses in this City, was found guilty and sentenced to two months' imprisonment with hard labour. The Mayor, who presided on the Bench, stated that the theft was a despicably mean one.

The University of Cambridge has acquired the copyright of the "Encyclopædia Britannica," and is about to publish, at the University Press, the eleventh edition of this well-known work. This edition is a completely new and original survey of human knowledge, down to the summer of 1910, and has, for the first time in the case of any first-class work of reference, been constructed as a whole, and will consequently be published as a whole.

The Medical Graduates' College and Polyclinic, 22 Chenies Street, W.C., reopens on Monday, September 12, and on Tuesday, September 13, Dr. J. E. Squire will give a Medical Clinique at 4 p.m., while other arrangements for the week are as follows: Wednesday, September 14, at 4 p.m., Mr. Edred M. Corner will treat with Surgery; Thursday, September 15, at 4 p.m., Dr. C. O. Hawthorne with Medicine; and Friday, September 16, at 4 p.m., Dr. William Hill will lecture on Ear, Nose, and Throat.

Mr. Cripps's former pupils at St. Bartholomew's have made him a presentation on the occasion of his retirement from the Hospital. A large number of gentlemen in all parts of the world subscribed. The presentation took the form of a solid silver bowl richly gilt, with caryatid handles, a copy of an old pattern of George II.'s time. On one side is engraved St. Bartholomew's crest, on the other "Presented to William Harrison Cripps, F.R.C.S., on his retirement from the office of Senior Surgeon to St. Bartholomew's Hospital, by members of his Surgical Classes, 1882 to 1909, as a token of esteem and regard, July, 1910." At the special request of Mr. Cripps the cup has been presented to him without ceremony.

Mr. Oddie held an inquest at Fulham last week on the body of Edwin Sidney Thomas, aged twenty-one years, a private in the 3rd Battalion Manchester Regiment. The deceased, who returned to his home at Fulham about a fortnight ago, complained on August 23 of pains in the head, and on the following day became unconscious. He was taken to the Fulham Infirmary, where he died. Dr. C. T. Parsons, the medical superintendent of the Infirmary, stated that, when admitted to the institution, the man was insensible, and suffering from brain fever, and that there was a tuberculous scar between some tattoo marks on his right arm. The tattooing had been carried out with Indian ink and a red pigment. Death was caused by tubercular meningitis, following inoculation with a germ of tubercle during the operation of tattooing. The jury returned a verdict of "Accidental death."

The St. Thomas's Hospital Old Students' Dinner will take place at the Hôtel Cecil on Tuesday, October 4, at 7 o'clock for 7.30. The chair will be taken by W. F. Haslam, Esq., F.R.C.S.

The distribution of prizes to the students of the Charing Cross Hospital Medical School will take place in the Out-patients' Hall at the Hospital (entrance in King William Street), on Monday, October 3, 1910, at 3.15 p.m., the Lady Juliet Duff will distribute the prizes. Tea and coffee will be served at the conclusion of the proceedings.

The Sixth London Medical Exhibition, organised by the *British and Colonial Druggist*, will be held in the first week in October at the Horticultural Hall, Westminster. The Exhibition will comprise all the latest novelties in medicine, hygiene, dietetics, surgical appliances, etc., and will be conducted on similar lines to former exhibitions which have been such pronounced successes. The whole arrangements will be strictly professional and of a high-class character. Invitation cards will be sent on September 29 to every member of the profession residing within a 20-mile radius of the Hall.

The Bombay Government has decided to apply the Lepers Act to many parts of the Presidency. Existing asylums will be enlarged, and, where necessary, new ones established. In this movement the authorities are co-operating with The Mission to Lepers in India and the East. Subsidised to a limited extent, the Mission is now segregating and supporting nearly 4,000 Indian lepers. A new asylum, built by the joint efforts of the Government and the Mission, was opened in Poona last year, with accommodation for 200 inmates, and is under the management of the latter. The experience of several years has convinced the authorities that both efficiency and economy are secured by co-operation with voluntary workers. At Sabathu, near Simla, in the Punjab, an old asylum is to be re-built and enlarged and placed under the control of the Mission. In addition to the Government share of the cost, the Society has to provide upwards of £500 towards building and equipment. At this station the Mission provides special accommodation for European and Eurasian lepers, and these will be duly considered in the new institution. Gifts towards meeting this need may be addressed to the Organising Secretary of the Mission, Mr. John Jackson, F.R.G.S., 33 Henrietta Street, London, W.C.

The Congress of the Royal Sanitary Institute, which met at Brighton this week, took into consideration a large number of interesting problems. In the Preventive Medicine Section, under the presidentship of Dr. Hope, of Liverpool, the economic effect of hygiene on the wage-earning capacity of the people was discussed, and infectious disease in connection with railway travelling was also dealt with, together with several subjects relative to the healthy administration of schools. In the Engineering and Architectural Sections, over which Mr. Henry Rofe, M.Inst.C.E., presided, the influence of subsoil waters on health was introduced by Mr Baldwin Latham, and several aspects of school planning, municipal baths and washhouses, and fever hospital construction came under consideration. The Conference of Municipal

Representatives, presided over by the Mayor of Brighton, dealt with the Notification of Births Acts, and the difficulties presented by the recent legislation with regard to midwives, the regulations recently imposed, although desirable in themselves, having shown up the fact that there are not, at present, sufficient midwives with the required qualifications to carry on the work. The most effective and economical means by which municipal authorities can provide sanatoria and treatment for the poorer members of the community was considered in the light of recent experience of the efforts that have been made by several authorities to meet this need. The Conference of Port Sanitary Authorities, presided over by Mr. Williamson, chairman, Port of London, considered the disinfection of ships in relation to the introduction of plague and cholera. For many years the careful measures taken at the ports of landing have prevented any serious outbreak in this country, but the continuance of this immunity is of such vast importance to the nation that a further consideration of these protective measures is just now of the greatest importance. In the Conference of Medical Officers of Health the registration of schools not controlled by the Board of Education was dealt with, the object being to provide some method by which proper space, ventilation, and general health conditions can be secured in private schools which at the present time have practically no supervision. The Women's Section, under the presidentship of the Countess of Chichester, devoted attention chiefly to papers on the feeding of infants and the teaching of practical hygiene in the home, and in the Childhood Section, under Sir William Collins, many aspects of child life were considered, including the feeble-minded child, and the measures that can be adopted for improving the condition of this unfortunate class, both in their own interests and the interests of others. The physical training of girls, which some people consider is likely to be carried too far, and the recent experience in treatment of ringworm by *x*-rays, were also included in the programme. The recent Housing and Town Planning Act, questions of sewage treatment, the development of roads for motor traffic, and many other matters of importance to health were brought forward at the meeting. The popular lecture was given by Dr. Alex. Hill, late Master of Downing College, on "The Bricks with which the Body is Built." "National Importance of Child Mortality" formed the subject of the lecture to the Congress by Dr. Arthur Newsholme.

